# Low fundamental and formant frequencies predict fighting ability among male mixed martial arts fighters

**DOI:** 10.1038/s41598-020-79408-6

**Published:** 2021-01-13

**Authors:** Toe Aung, Stefan Goetz, John Adams, Clint McKenna, Catherine Hess, Stiven Roytman, Joey T. Cheng, Samuele Zilioli, David Puts

**Affiliations:** 1 Department of Anthropology, The Pennsylvania State University, 409 Carpenter Building, University Park, PA 16802 USA; 2 Wayne State University, Detroit, USA; 3 University of Michigan, Ann Arbor, USA; 4 York University, Ontario, Canada

**Keywords:** Sexual selection, Anthropology, Biological anthropology, Psychology, Human behaviour

## Abstract

Human voice pitch is highly sexually dimorphic and eminently quantifiable, making it an ideal phenotype for studying the influence of sexual selection. In both traditional and industrial populations, lower pitch in men predicts mating success, reproductive success, and social status and shapes social perceptions, especially those related to physical formidability. Due to practical and ethical constraints however, scant evidence tests the central question of whether male voice pitch and other acoustic measures indicate actual fighting ability in humans. To address this, we examined pitch, pitch variability, and formant position of 475 mixed martial arts (MMA) fighters from an elite fighting league, with each fighter’s acoustic measures assessed from multiple voice recordings extracted from audio or video interviews available online (YouTube, Google Video, podcasts), totaling 1312 voice recording samples. In four regression models each predicting a separate measure of fighting ability (win percentages, number of fights, Elo ratings, and retirement status), no acoustic measure significantly predicted fighting ability above and beyond covariates. However, after fight statistics, fight history, height, weight, and age were used to extract underlying dimensions of fighting ability via factor analysis, pitch and formant position negatively predicted “Fighting Experience” and “Size” factor scores in a multivariate regression model, explaining 3–8% of the variance. Our findings suggest that lower male pitch and formants may be valid cues of some components of fighting ability in men.

## Introduction

Sexually dimorphic vocalizations appear to have been shaped by sexual selection across a variety of species, including Australian field crickets (*Teleogryllus oceanicus*)^1^, túngara frogs (*Physalaemus pustulosus*)^2^, common loons (*Gavia immer*)^3^, red deer (*Cervus elaphus*)^4^, chacma baboons (*Papio ursinus*)^5^, and other anthropoid primates^6^, including humans (*Homo sapiens*)^7^. Pitch is the most perceptually salient acoustic characteristic of the human voice and is determined primarily by fundamental frequency (*f*_o_), the rate of vocal fold vibration during phonation^8^. Pubertal increases in circulating testosterone and abundant androgen receptors in the vocal folds^9^ cause male vocal folds to grow approximately 60% longer than those of women^8^. Consequently, men’s vocal folds vibrate at lower frequencies, and men speak at approximately half the *f*_o_ of women^6^, a difference of about five standard deviations^10^.

Cross-species comparison suggests that sexual dimorphism in *f*_o_ is likely to evolve under conditions of intense male-male competition in anthropoid primates^6^. Among humans living in both traditional and industrial populations, lower male *f*_o_ predicts mating success, reproductive success, and social status^11^ and conveys the impression that the speaker is more physically formidable^7,12–16^. Because low *f*_o_ reliably distinguishes adult males from adult females and children, it is a valid cue of physical formidability among humans generally^17^. More puzzling, however, is the abundance of evidence suggesting that listeners readily use *f*_o_ as a cue for inferring a variety of traits even *within* sex, attributing greater size, strength, and formidability to adult male voices with lower *f*_o_^12,14,18,19^. Yet, scholars debate the accuracy of these perceptions^11,17,18,20,21^. According to one hypothesis, attention to men’s *f*_o_ is the perceptual by-product of a broader tendency to associate low-frequency sounds with physically larger sources. Hence, listeners may perceive low frequencies to be intimidating even when *f*_o_ is unrelated to the size of the sound producer^18,21^. However, lower *f*_o_ has been found to predict men’s status, as well as mating and reproductive success^17^. If these benefits were obtained partly through the influence of *f*_o_ on social impressions, then deferring to men with low *f*_o_ appears to have important costs that could be compensated only if there is an adaptive advantage to doing so^11,17^. Selection should favor inattention to individual differences in men’s *f*_o_ unless the signal is at least partly honest^22,23^.

Previous studies have investigated the information content of *f*_o_ by exploring relationships with perceived formidability and its correlates such as size, strength, and hormonal concentrations. Participants can rapidly and accurately assess strength from men’s voices^19,24^. Although some studies have reported null findings between voice pitch and measures of strength^25–28^, testosterone^27,29,30^, and health^31^ among men, meta-analyses indicate that low *f*_o_ in men predicts greater height (*r* ~ − 0.2)^32^, testosterone concentrations (*r* ~ − 0.2)^17^, and upper-body strength (*r* ~ − 0.1)^17^. Although the strengths of these relationships are notably weak, each measure is an imperfect correlate of formidability, and any relationship between *f*_o_ and overall formidability would be attenuated by error in quantifying formidability with a single correlate such as height. In addition, associations between *f*_o_ and testosterone were found to be stronger among men with lower cortisol levels^6,33^, a pattern of hormonal associations that may reflect greater immunocompetence^34^. Lower male *f*_o_ has been reported to be associated with elevated mucosal immunity^29^ and energetic condition^35^.

Despite the growing interest in the human male voice as a target of sexual selection, scant evidence directly tests the central question raised by the strong effects of *f*_o_ on perceived formidability: Does low *f*_o_ predict actual ability in physical confrontations among men? One means of circumventing the practical and ethical constraints on addressing this question is by utilizing data from men who engage in fighting for sport. In the only previous study of this type of which we are aware^28^, vocal *f*_o_ did not predict win percentage among 29 male mixed martial arts (MMA) fighters. In this small group of fighters, *f*_o_ was obtained from a standardized passage (i.e., counting from 1 to 10) recorded on the day prior to competing in the IMMAF European Open Championships. This study was underpowered for detecting what is likely to be a small to moderate effect. Difficulties inherent to quantifying small differences in fighting ability among the upper echelons of competitive fighters, as well as naturally occurring within-individual variability in acoustic parameters across contexts^13,16,36–38^, means that any strong empirical tests and efforts to precisely estimate effect size will require large samples, combined with multiple measures of both fighting ability (i.e., beyond win percentages) and acoustic parameters of interest.

In the current pre-registered study, we obtained 1312 voice samples from interviews of 475 MMA fighters from an elite fighting league. From these samples, we measured *f*_o_ and several other sexually dimorphic acoustic parameters that have previously been linked with dominance, including *f*_o_ variability (*f*_o_-SD), formant dispersion (*D*_*f*_), and formant position (*P*_*f*_)^10,32,39^. Lower *f*_o_-SD indicates a more monotone voice, and both *D*_*f*_ and *P*_*f*_ are inversely related to vocal tract length and negatively influence perceptions of vocal timbre. *D*_*f*_ measures the average distance between successive formant (resonant) frequencies (in Hz) for the first *n* (usually 4) formants, whereas *P*_*f*_ is the average standardized formant value for the first *n* (usually 4) formants and measures how high or low formants are on average in standard deviations from the sample mean^10^. We tested the pre-registered hypotheses that lower, more male-typical *f*_o_, *f*_o_-SD, *D*_*f*_, and *P*_*f*_ predict prevailing in physical confrontations, defined as fighting success indexed by fight outcomes and statistics across multiple physical encounters. We adhered to our pre-registered plans and conducted analyses using both *D*_*f*_ and *P*_*f*_. However, at the request of a reviewer, we report only *P*_*f*_ as the formant measure in our main manuscript, as *D*_*f*_ is a less precise measure of formant structure^10,32,40^. Results involving *D*_*f*_ can be found in our result output document available online. In our initial pre-registration, we planned to assess fighting ability via three measures of fighting success outcomes: total number of fights, Elo rating, and retirement status. However the reasons outlined below in our exploratory analyses section (i.e., fighting ability is likely a multidimensional construct), such separate measures are unlikely to fully capture fighting ability. Thus, we went beyond our pre-registered plans to additionally extract distinct dimensions of fighting ability via factor analysis of all fighting-related measures obtained in our study (height, weight, years active, age, total number of fights, Elo rating, retirement status, and win percentage) and explore how these dimensions were associated with *f*_o_ and other acoustic measures.

## Results

### Pre-registered analyses

In multilevel models with individual acoustic measures as sole predictors (Fig S1), lower *f*_o_ (*b* = − 0.11*, p* = 0.010), *f*_o_-SD (*b* = − 0.11*, p* = 0.021), and *P*_*f*_ (*b* = − 0.15*, p* < 0.001) significantly predicted total number of fights, only *P*_*f*_ (*b* = − 0.15*, p* = 0.030) predicted Elo ratings, and no acoustic measures predicted retirement status (Table S2). Zero-order correlations between best linear unbiased estimates of acoustic parameters and measures of fighting success produced similar results (Table 1). When all four acoustic measures and covariates, such as height and weight, were entered simultaneously as predictors of fighting success (including win percentage, an exploratory, non-pre-registered variable), none reached conventional levels of significance, indicating that acoustic measures did not explain unique variance over and above each other (Table S3). This is perhaps unsurprising given that some acoustic measures are positively correlated and thus share some degree of overlapping variance (see Table 1).

**Table 1:** Zero-order correlations among study variables.

Variable status	*f*_o_	*f*_o_-SD	*P*_*f*_	Height	Weight	Active years	Age	Total fights	Elo rating	Retirement
*f*_o_-SD	0.48***									
*P*_*f*_	0.24***	0.21***								
Height	− 0.18***	− 0.10*	− 0.24***							
Weight	− 0.15**	− 0.08	− 0.27***	0.74***						
Active years	− 0.10*	− 0.10*	− 0.08*	0.08	0.17***					
Age	− 0.17***	− 0.09	− 0.20***	0.11*	0.30***	0.30***				
Total fights	− 0.11*	− 0.09*	− 0.13**	0.11*	0.21***	0.73***	0.44***			
Elo rating	− 0.04	− 0.03	− 0.11*	0.16**	0.23***	0.47***	0.15**	0.41***		
Retirement status	− 0.04	− 0.01	− 0.03	0.07	0.05	− 0.31***	0.20***	0.09	− 0.22***	
Win percentage	− 0.03	− 0.01	− 0.05	0.02	0.03	0.31***	0.03	0.20***	0.37***	− 0.37***

Acoustic variables are computed as the best linear unbiased measures for multiple recordings measured at the individual level by using micro-macro multi-level modeling^73^, which is preferred to using simple group means^74^.

*p* < .05*, *p* < .01**, *p* < .001 ***.

### Exploratory analyses

**Figure 1:**
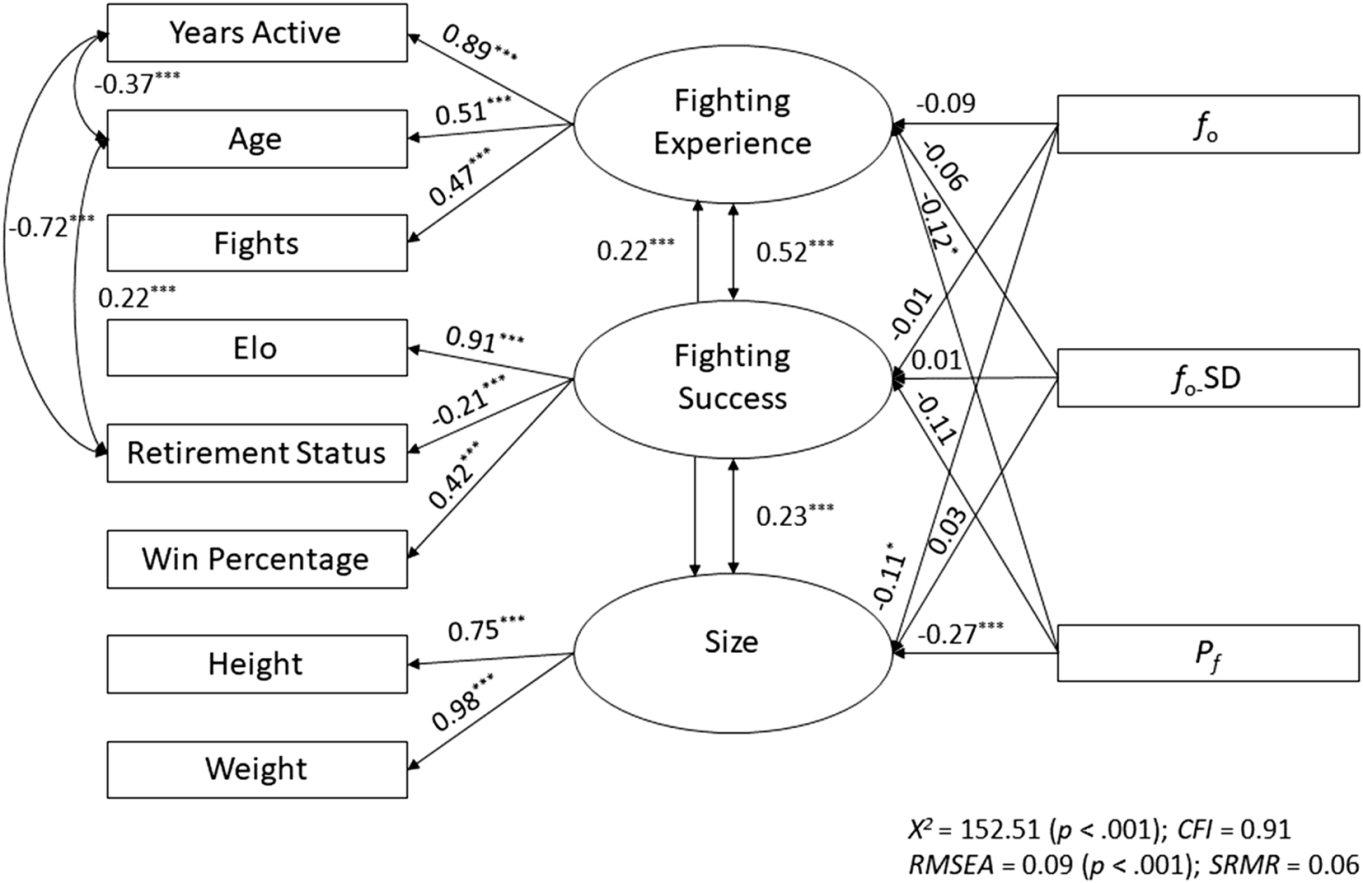
Path diagram for a structural equation model highlighting standardized associations among all observed (rectangular) and latent (oval) variables. We first fitted an orthogonal model, but allowing residual correlations between latent variables significantly improved the model (*p* < .001). Note. *f*_o_ = fundamental frequency; *f*_o_-SD = Variability in fundamental frequency; *P*_*f*_ = formant position; *X*^2^ = model chi square; CFI = comparative fit index; RMSEA = root mean square error of approximation; SRMR = standardized root mean square residual. *p* < .05^*^, *p* < .01^**^, *p* < .001 ^***^.

Two key issues likely complicated our initial pre-registered assessments of fighting ability. The first issue is that although single measures of fighting ability, such as Elo ratings and win percentage, are correlated (*r* = 0.37; Table 1) and thus internally consistent, there are limitations to each; for example, fighters with no losses have a win percentage of 100% regardless of their number of fights. To address this issue, we conducted a principal axis factoring to reduce the set of eight fighting ability-related measures (height, weight, years active, age, total number of fights, Elo rating, retirement status, and win percentage) into dimensional factors (*n* = 3) using the “nFactors” package^41^, and rotated factors with the Varimax method using the “psych” package^42^. As shown in Table 3, onto Factor 1 (which we label “Fighting Experience”), number of fights, years active, age, and Elo rating loaded most strongly. Onto Factor 2 (“Fighting Success”), retirement status, win percentage, Elo rating, and years active loaded most strongly. Finally, onto Factor 3 (“Size”), height and weight loaded most strongly. Factor scores on each of these 3 factors were then used as dependent variables, in separate regression models, with all acoustic measures as predictors (Table 2). Results from these models indicate that *P*_*f*_ negatively predicted Fighting Experience, whereas *f*_o_ and *P*_*f*_ negatively predicted Size (Fig S2). To strengthen our findings, we also conducted a structural equation model that takes measurement error into account and obtained the same pattern of results and acceptable model fit (Fig. 1).

**Table 2:** Results of micro–macro multi-level models predicting three component measures of fight metrics from acoustic measures.

Predictors	Factors		
	Fighting Experience	Fighting Success	Size
Intercept	− 0.01 (.903)	0.01 (.976)	− 0.01 (.966)
*f*_o_	− 0.10 (.059)	0.04 (.405)	− 0.13 (.018)
*f*_o_*-*SD	− 0.04 (.507)	− 0.04 (.633)	0.04 (.534)
*P*_*f*_	− 0.20 (.002)	0.02 (.832)	− 0.32 (< .001)
*R*^2^	0.04	0.01	0.08

Results are reported as effect size (p-value). Effect sizes are beta-weights, except for retirment status, which is odds ratio. In all models, the variance inflation factor (VIF) of each predictor was < 1.5. *f*_o_ = fundamental frequency; *f*_o_-SD = Variability in fundamental frequency; *P*_*f*_ = formant position.

**Figure 2:**
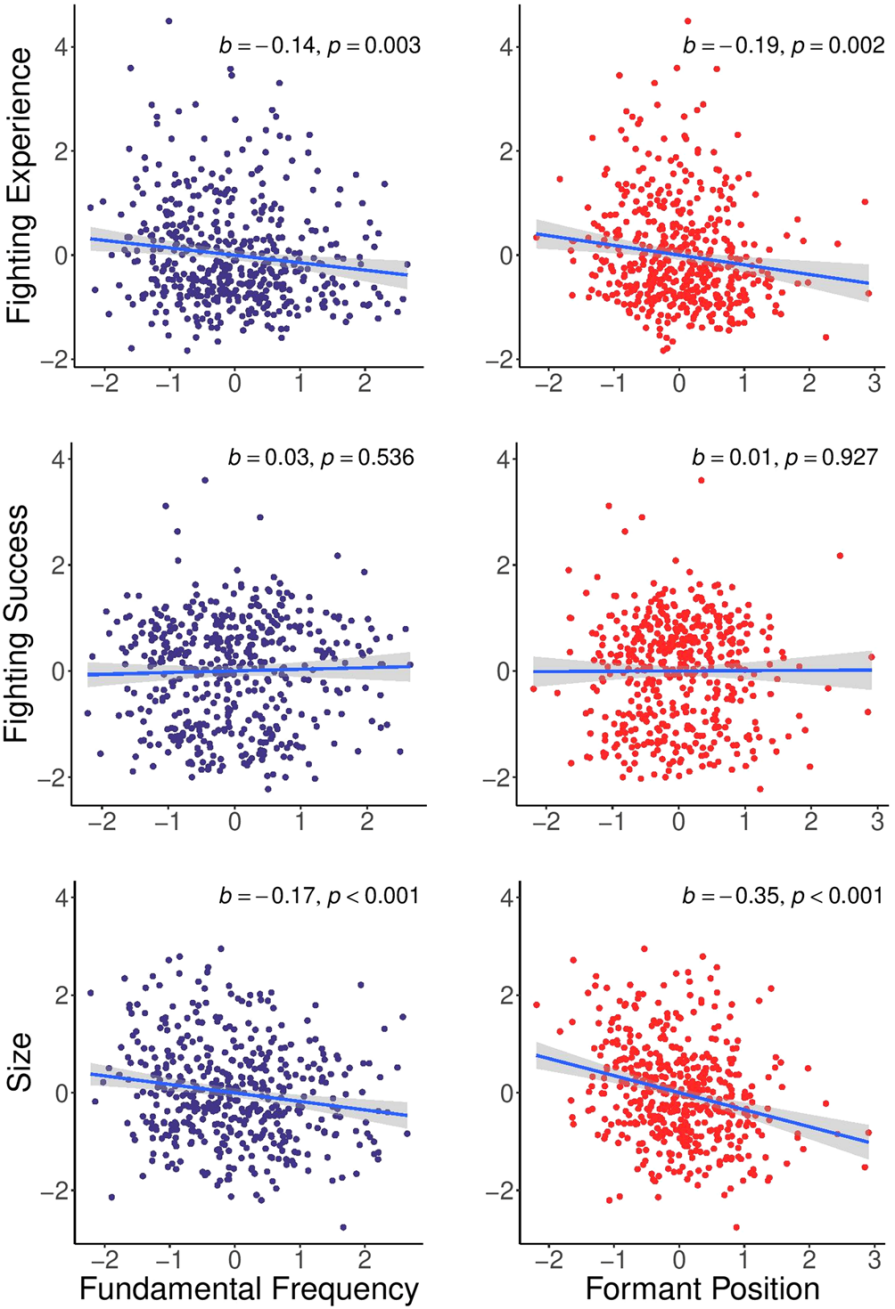
Relationships between fighting ability components and acoustic measures. The relationship between fundamental frequency (left column) and formant frequency (right column) are plotted against Fighting Experience (top row), Fighting Success (middle row), and Size (bottom row).

A second issue, highlighted by our principal axis factoring, is that fighting ability is multidimensional. Although Fighting Experience explained the most variance in fighting ability-related variables (27%), Fighting Success (23%) and Size (22%) also explained substantial proportions and captured unique components of fighting ability. For example, fighters compete only against opponents within the same weight class. Body size is sufficiently decisive in fights to necessitate weight classes; a 52.2 kg strawweight fighter and a 120.2 kg heavyweight fighter with identical Elo ratings, win percentage, and so forth are not equivalent in fighting ability, for instance. To test whether fighting ability relates to an acoustic parameter such as *f*_o_, it is important to consider all major components of fighting ability simultaneously. Although the structural equation model reported above considered all three extracted components of fighting ability, structural equation models test association, not significance, as their main function is to evaluate whether the model conforms to the data^43^. To test whether *f*_o_ and other acoustic parameters predict overall fighting ability, we therefore conducted a multivariate regression model where Fighting Experience, Fighting Success and Size were simultaneously entered as dependent variables and acoustic measures as predictors (Table 4). We observed statistically significant relationships between fighting ability and both *f*_o_ and *P*_*f*_, but not *f*_o_*-SD*. When we compared the multivariate model with all acoustic measures to the model with only *f*_o_ and *P*_*f*_ as predictors, we found that the latter model performed as well as the former. Both *f*_o_ and *P*_*f*_ negatively predicted Fighting Experience and Size but neither predicted Fighting Success. We obtained similar results when *f*_o_ and *P*_*f*_ were entered as single predictors in separate multivariate regression models (Fig. 2).

## Discussion

Sexual dimorphism in *f*_o_ likely arose in the common ancestor of the catarrhine primates after their divergence from the New World monkeys^6^ approximately 43.5mya^44^ and appears to have been subsequently elaborated or reduced depending on the form and degree of male mating competition^6^. Relatively low male *f*_o_ may have evolved as a means of exaggerating the appearance of size to same-sex competitors and/or potential mates^6,18^, but there is considerable debate regarding whether male *f*_o_ is purely deceptive^20,21,45^ or provides any reliable information about formidability in men^11,17,33^.

To shed light on this debate, we investigated whether *f*_o_ is associated with fighting ability among a large sample of male MMA fighters. Results of our pre-registered analyses were generally in the direction of lower *f*_o_ predicting greater fighting ability but were mixed in terms of statistical significance. When we addressed the limitations of these analyses by creating more precise measures of fighting ability and accounting for the contributions of the distinct dimensions revealed through principal axis factoring, we found that lower *f*_o_ was associated with greater fighting ability in all analyses. In the statistical model that most precisely measured fighting ability by including principal axis factors related to fighting experience, fighting success, and body size, *f*_o_ predicted fighting ability generally and specifically components of fighting ability related to experience and size, but not within-weight class fighting success. Overall, these results suggest that low *f*_o_ is an honest cue of formidability in men (Table 3).

**Table 3:** Component axis analysis for measures related to fighting ability.

Predictors	Factors		
	Factor 1: Fighting Experience	Factor 2: Fighting Success	Factor 3: Size
Number of fights	**0.90**	0.13	
Years active	**0.74**	**0.48**	
Age	**0.72**	− 0.27	0.16
Elo rating	**0.44**	**0.56**	0.21
Weight	0.19		**0.91**
Retirement status	0.19	**− 0.81**	
Win percentage	0.15	**0.73**	
Height			**0.93**
Variance explained	0.27	0.23	0.22

Loadings that were above |0.4| are bolded. Factors are named descriptively according to the variables that load most strongly onto them. Empty cells indicate factor loadings <|0.1|.

Effect sizes were small, however. Even when fighting ability was measured most precisely, *f*_o_ explained only 1–3% of the variance (Table 4). On the one hand, the strength of these associations accords with theoretical predictions derived from the fact that signaling is multimodal and multi-component^17^. On the other hand, it is important to emphasize that relationships between *f*_o_ and fighting success among MMA fighters may underestimate those in the general population due to range restriction on fighting ability. Among elephant seals, the body length of males who occupied the center of harems correlated with neither maximum harem size nor tenure length on the beach during the breeding season^46^. However, when all males were analyzed together, including those that were peripheral to or outside of the harem, male body length explained 17% of the variation in tenure length on the beach. The degree to which *f*_o_ correlates with fighting ability in the general population has yet to be tested for obvious ethical reasons.

**Table 4:** Results of a multi-variate regression model.

	Fighting Experience	Fighting Success	Size	Pillai test
**Model 1**				
*f*_o_	− 0.10 (.903)	0.05 (.429)	− 0.13 (.017)	0.02 (.019)
*f*_o*-*_SD	− 0.04 (.577)	− 0.04 (.604)	0.04 (.536)	0.01 (.819)
*P*_*f*_	− 0.20 (.005)	0.02 (.829)	− 0.32 (< .001)	0.06 (< .001)
*R*^*2*^	0.04	0.01	0.08	
**Model 2**				
*f*_o_	− 0.11 (.022)	0.03 (.539)	− 0.12 (.018)	0.02 (.008)
*P*_*f*_	− 0.15 (.016)	− 0.01 (.954)	− 0.31 (< .001)	0.07 (< .001)
*R*^*2*^	0.03	0.01	0.08	
**Model 3**				
*f*_o_	− 0.14 (.003)	0.03 (.536)	− 0.17 (< .001)	0.05 (< .001)
*R*^*2*^	0.02	0.01	0.03	
**Model 4**				
*P*_*f*_	− 0.19 (.002)	0.01 (.927)	− 0.35 (< .001)	0.09 (< .001)
*R*^*2*^	0.02	0.01	0.07	

Results are reported as effect size (p-value). Effect sizes are beta-weights, except for Pillai’s trace test statistics. *f*_o_ = fundamental frequency; *f*_o_-SD = Variability in fundamental frequency; *P*_*f*_ = formant position.

Voice pitch and other acoustic variables are modulated across social contexts, including those related to perceived fighting ability relative to a competitor^13,47^, relative dominance and prestige^48^, authority^36^, current aggressive intent^49^, emergent rank^16^, and volitional exaggeration^50,51^. On the one hand, if voice pitch is modulated in relation to self-perceived relative formidability and status, as this prior research indicates, then it is possible that any voice modulation of fighters during interviews could have strengthened relationships between acoustic parameters and measures of fighting ability in the present study. On the other hand, if voice modulation is less patterned or less dependent upon perceived relative formidability, then this would introduce noise in measuring individual differences in voices, which would tend to weaken relationships between acoustic parameters and true fighting ability. In these data based on naturalistic observations, although we cannot rule out that some fighters may have modulated their voice pitch or other vocal parameters during interviews to sound stronger, we sought to strengthen our measures of individual differences in acoustic traits by sampling across multiple interview occasions. Indeed, we found that acoustic measures were consistent across fighters, even between pre- and post-fight conditions in the subset of our sample for which this information was available (see Supplemental Procedures for details), and we attempted to capture more stable individual acoustic differences rather than measurements specific to particular recording conditions by using the unbiased linear estimates across recordings for each fighter in all analyses.

Only one previous study of which we are aware^28^ examined links between fighting success and *f*_o_ or other acoustic measures and found that acoustic measures did not predict win percentage among male MMA fighters. Likewise, in our bivariate correlation analysis, we did not find a significant relationship between win percentage and vocal *f*_o_ (Table 1), although lower *f*_o_ predicted a greater number of UFC matches. Our ability in subsequent analyses to detect relationships between *f*_o_ and fighting ability where a previous study did not^28^ may have been due in part to our examination of a larger sample of fighters (474 vs. 29), along with longer total voice samples from each fighter (approximately 85 s vs. 8 s).

Our use of unstandardized, spontaneous speech samples also offered enhanced ecological validity^47^, and although this approach adds noise to the measurement of individual differences in acoustic parameters, previous research shows strong correspondence across speech and/or vocalization types for both fundamental^52^ and formant^10^ frequencies. Moreover, the best linear unbiased composite estimates of acoustic measures from multiple voice recordings of each fighter, along with relatively long (> 85 s) average total speech samples from individual fighters, provided reliable estimates of individual differences in acoustic features.

Perhaps most importantly, we extracted component variables via factor analysis to produce more comprehensive measures of fighting ability than can be offered by individual measures such as Elo rating or win percentage. For example, win percentage alone is an imperfect indicator of fighting success because fighters with fewer fights and losses can achieve higher win rates^53,54^ and because win percentage does not account for the strength of opponents as Elo ratings do. Our factor analysis addressed such limitations and produced three unique components of fighting ability, which we termed Fighting Experience, Fighting Success, and Size. Fighting Experience reflects the component of flighting ability most strongly related to total number of fights but also strongly related to years active in the UFC and age. Given that Elo ratings also loaded positively onto this component, it may represent the component of fighting skill attributable to experience. Fighting Success reflects a component of fighting ability related to a history of winning in one’s weight class that is relatively unrelated to experience or size, perhaps tapping characteristics such as speed, agility, and strength for one’s size. The extracted Size factor reflects the component of fighting ability related to height and weight. Larger size is generally associated with lower *f*_o_ across mammals^55^ and is a major determinant of dominance and social status across species^56–60^. Physical size is such a strong determinant of outcomes in combat sports such as wrestling, boxing, and martial arts that weight classes are needed to prevent dangerous lopsided contests, and fighters are willing to sacrifice energy and hydration by cutting weight to fight smaller opponents. Parallel to findings in previous studies^32,61^, fighters’ *f*_o_ predicted their height and weight, as well as their Size factor scores.

Our orthogonal factor analysis extracted independent components of fighting ability, maximizing the variance among items and facilitating interpretation. A structural equation model further confirmed the structure of our proposed components of fighting ability, but allowing residual correlations between latent variables produces a better model fit. These analyses highlight the importance of considering fighting ability across related measures instead of using a single measure of fighting experience, fighting success, or size. Because fighting ability measures (e.g., win percentage and number of fights) reflect weight division-specific measures, results from analyses that do not incorporate the influence of fighter’s weight (Table 1) may be less valid than others (Table 4).

Although not the focus of the present paper, other sexually dimorphic acoustic parameters were associated with fighting ability. In correlation analyses, lower *f*_o_-SD and *P*_*f*_ predicted a greater number of UFC fight matches, and lower *P*_*f*_ predicted higher Elo ratings (Table 1). In addition, *P*_*f*_ predicted overall measures of fighting ability in the multivariate regression model (Fig. 2) and independently predicted Fighting Experience and Size (Fig. S2). Our findings extend those from previous studies showing that *P*_*f*_ predicts objective measures of threat potential, such as size^32^, strength, and physical aggression^10^, as well as perceived fighting ability^14,62^, and suggest that *P*_*f*_ communicates size-independent information about vocalizers’ physical formidability.

A potential statistical issue concerns the use of uncorrected *p*-values associated with multiple tests. However, the adjustment of *p*-values, such as Bonferroni correction, not only decreases the rates of Type I error, but also increases the rates of Type II error and has been highly criticized^63^. Additional analyses used in this study, such as multi-level modeling^64^, multi-variate regression, and structural equation modeling, that consider dependent variables simultaneously should reduce concerns associated with multiple comparisons.

In general, our findings are consistent with the broader perspective that men’s anatomy, behavior, and psychology have been shaped by an evolutionary history of contest competition^27,65^, the use of force or threat of force to exclude same-sex competitors from mates^59^. Contest competition should favor psychological mechanisms to attend to and assess the formidability and threat potential of competitors^19^. People can accurately assess physical strength from body^19^ and face^66^ images. For example, sexually dimorphic facial cues predict fighting ability among MMA fighters^33,67^ but see^68^, and using these cues participants can predict fighting outcomes above chance^69^. The voice appears to be another aspect of the phenotype that indicates formidability^10,13,19^; we showed that *f*_o_ was associated with both body size and fighting experience (independent of body size) among male MMA fighters. Although associations between measures of fighting ability and *f*_o_ and other sexually dimorphic acoustic parameters were small, they comport with the notion that *f*_o_ is but one of many components of a vocal acoustic signal, and that voices represent one of many signals of men’s formidability^17^. A deep, resonant voice clearly indicates status as a physically mature or maturing male. Our findings support the further possibility that attention to differences in voice pitch among men may be functional as well, as *f*_o_ and other sexually dimorphic components of men’s voices appear to provide information about differences in men’s threat potential.

## Method

### Samples

Out of all fighters in the MMA promotion company Ultimate Fighting Championship (UFC) who were active up to the end of 2013 (UFC event 168, 12/28/2013), we obtained data on 475 fighters for our analyses (see Supplemental Procedures for details). Data were not collected after 2013, as there was a spike in the number of UFC events in 2014 that may have confounded results. Following prior work^54^, we included only experienced fighters, defined as those who have fought at least 10 professional fights, at least one of which was in the UFC.

### Fighting ability measurement

We obtained fighters’ age, height, weight, total number of fights, and total number of wins from Wikipedia and Sherdog websites. Because, during the sampling period, fighters who experienced three consecutive losses were likely eliminated from the UFC^70^, only relatively successful fighters were likely to remain in the UFC. To measure long-term fighting ability, we obtained the retirement status (whether the fighter retired between 2013 and 2018) and calculated the total number of years a fighter was active (the total number of years between the fighter’s first UFC fight and his last UFC fight between 2013 and 2018). In addition, we obtained Elo-equivalent ratings as another measure of fighting ability for each fighter. These are computerized objective rankings generated by the FightMatrix website that utilizes a proprietary engine (CIRRS – Combat Intelli-Rating and Ranking System) and a comprehensive MMA fight database to adjust ratings according to each fighter’s wins and losses and the records of opponents. For descriptive statistics on fighting ability measures, see Table S1.

### Acoustic measurement

To provide a more precise measure of person-level *f*_o_, we attempted to collect multiple (up to five) voice samples from each fighter samples (range = 1–5, *M* = 2.76, *SD* = 1.15) from unique audio and video interviews obtained from YouTube, video.google.com, or podcasts from radio shows and downloaded as .wav files. We prioritized pre-fight recordings but also obtained post-fight recordings, identifying them accordingly in our dataset. Secondary sounds (e.g., background noise, interviewers’ voices) were removed from downloaded recordings so that only the voice of each fighter was extracted. A mean of 30.99 s (range = 4–594.98, *SD* = 55.62) of a fighter’s voice was extracted from each interview, so that the combined length of all voice samples from a fighter averaged 85.32 s (range = 5.43–1099.33, *SD* = 133.75). We then used Praat to measure *f*_o_, *f*_o_-SD, *D*_*f*_, and *P*_*f*_ from each voice sample. For mean *f*_o_ and *f*_o_-SD, pitch floor was set to 75 Hz, and pitch ceiling was set to 300 Hz in accordance with programmer recommendations. Otherwise, default settings were used. For *D*_*f*_ and *P*_*f*_ calculations^10^, *f*_1_ through *f*_4_ were measured at each glottal pulse (automated detection by Praat) and averaged across measurements. Before analyses, we removed one fighter from our sample whose *P*_*f*_ measurement was 7.5 SD above the mean, a decision not specified in our pre-registration document. Our final sample comprised 1311 recordings from 474 fighters (see Supplemental Procedures for details).

## Data analysis

Most common multilevel models focus on macro–micro conditions; a mix of both lower micro-level (level 1) and higher macro-level (level 2) explanatory variables are used to predict lower micro-level (level 1) outcomes^71^. However, our data diverge from this typical condition, as they exhibit the opposite micro–macro pattern, with our acoustic measures predictors collected multiple times for (that is, nested within) the same fighter (level 1), and our dependent variables (e.g., fighting ability) captured for each fighter (level 2). Given this somewhat anomalous feature of our data, predicting level 2 variables from level 1 variables using standard multilevel techniques would produce statistically biased results^72^. To circumvent these issues, we used the “MicroMacroMultilevel” package^73^ in *R* that allows micro–macro multi-level models^74^ by producing the best linear unbiased predictors (BLUPs) for all group aggregates of variables measured at the lowest level. All acoustic measures and covariates, including Elo ratings, were z-scored before analyses (see Table 1 for correlations among study variables), so all results reported below represent standardized effects. All analyses were conducted using R 3.6.1. Three models in our pre-registered tests were estimated, each with a different dependent variable—a given fighter’s total number of fights, Elo ratings, and retirement status. For each dependent variable, the BLUP of the group mean for each acoustic measure was entered as the sole predictor in our multi-level models. In subsequent models, all four BLUPs of the group mean for acoustic measures were simultaneously entered as predictor variables, and fighters’ height, weight, age, and number of active years were entered as additional level 2 covariates. Detailed methods for additional tests are described above. Data and scripts for all analyses are made available online (see Supplemental Procedures).

### Ethics

Data were obtained from online sources that are are freely available to public.

## Supplementary information

Supplementary Information 1.

## Data Availability

The raw data and scripts for all our models are also made available online at https://osf.io/md6wj/?view_only=81cf6446a90448a594e1e1ec6b25ce59.
