# Supplementary material for: Low fundamental and formant frequencies predict fighting ability among male mixed martial arts fighters
Source: Sci Rep. 2021 Jan 13;11:905. doi: 10.1038/s41598-020-79408-6 (PMC7806622; doi:10.1038/s41598-020-79408-6)
Supplement: Supplementary file 1 — Supplementary Information 1. [file 41598_2020_79408_MOESM1_ESM.docx]

**Low fundamental and formant frequencies predict**

**fighting ability among male mixed martial arts fighters**

Toe Aung,^1^ Stefan Goetz,^2^ John Adams,^2^ Clint McKenna,^3^

Catherine Hess,^1^ Stiven Roytman,^2^ Joey T. Cheng,^4^ Samuele Zilioli,^2^ and David Puts^1 *^

^1^ Pennsyvlania State University

^2^ Wayne State University

^3^ University of Michigan – Ann Arbor

^4^ York University

*Corresponding author:
David Puts

409 Carpenter Building

Department of Anthropology

The Pennsylvania State University

University Park, PA, 16802

USA

Email: dap27@psu.edu

**Table S1.** Descriptive statistics.

| **Fighting-related measures** | **Mean** | **SD** | **Range** |
| --- | --- | --- | --- |
| Height (m) | 1.79 | 0.08 | 1.5-2.01 |
| Weight (kg) | 77.49 | 14.65 | 56.7-129 |
| Active years | 4.61 | 3.73 | <1-21 |
| Age | 35.57 | 4.18 | 25-55 |
| Total fights | 5.88 | 5.12 | 1-26 |
| Elo rating | 157.09 | 175.98 | 21-1547 |
| Retirement status (1 = retired) | 0.31 | 0.46 | 0-1 |
| Win percentage | 0.52 | 0.31 | 0-1 |
| **Acoustic measures**  **(all recordings, with multiple recordings per fighter)** | **Mean** | **SD** | **Range** |
| *f*_o_ | 122.78 | 16.15 | 82.01-165.81 |
| *f*_o_*_-_*SD | 19.84 | 7.2 | 3.83-70.74 |
| *D_f_* | 995.63 | 55.82 | 720.62-1185.45 |
| *P_f_* | 0 | 0.65 | -1.84-7.91 |
| **Fighter-level acoustic measures (between fighters)** | **Mean** | **SD** | **Range** |
| *f*_o_ | 122.46 | 15.25 | 86.99-165.59 |
| *f*_o_*_-_*SD | 19.73 | 5.62 | 5.76-49.15 |
| *D_f_* | 997.28 | 48.81 | 831.76-1178.93 |
| *P_f_* | 0.02 | 0.56 | -1.44-6.08 |
| **Average difference in acoustic measures (within fighters)** | **Mean** | **SD** | **Range** |
| *f*_o_ | 7.33 | 4.88 | 0.35-46.48 |
| *f*_o_*_-_*SD | 5.08 | 4.73 | 0.03-34.99 |
| *D_f_* | 34.34 | 26.85 | 0.23-201.02 |
| *P_f_* | 0.36 | 0.33 | 0.01-2.73 |

Note. *f*_o_ = fundamental frequency; *f*_o_-SD *= ­*Variability in fundamental frequency; *D_f_* = formant

dispersion; *P_f_* = formant position. Average difference in acoustic measures within fighters was

calculated by the (x_n_-x_1_) / (n -1).

**Table S2.** Multilevel models with each acoustic measure as a predictor.

|  | **Number of fights** | **Elo ratings** | **Retirement status** | **Win percentage** |
| --- | --- | --- | --- | --- |
| *f*_o_ | -0.11 (.010) | -0.04 (.381) | 0.91 (.344) | -0.01 (.482) |
| *f*_o_*_-_*SD | -0.11 (.021) | -0.04 (.607) | 1.00 (.978) | -0.01 (.907) |
| *D_f_* | -0.01 (.930) | -0.08 (.161) | 1.01 (.912) | 0.01 (.871) |
| *P_f_* | -0.15 (< .001) | -0.15 (.030) | 0.92 (.579) | -0.02 (.290) |

Note. Results are reported as effect size (p-value). Effect sizes are beta-weights, except for retirment status, which is odds ratio. *f*_o_ = fundamental frequency; *f*_o_-SD *= ­*Variability in fundamental frequency; *D_f_* = formant dispersion; *P_f_* = formant position; *OR* = odds ratio.

|  | **Number of fights** | | **Elo ratings** | | **Retirement status** | | **Win percentage** | |
| --- | --- | --- | --- | --- | --- | --- | --- | --- |
| **Predictors** | Model 1 | Model 2 | Model 1 | Model 2 | Model 1 | Model 2 | Model 1 | Model 2 |
| Intercept | 1.76 (<.001) | 1.62 (<.001) | 0.01 (.958) | 0.01 (.965) | 0.45 (<.001) | 0.35 (<.001) | 0.52 (<.001) | 0.52 (<.001) |
| *f*_o_ | -0.07 (.156) | -0.01 (.976) | -0.01 (.821) | 0.02 (.583) | 0.89 (.327) | 0.96 (.805) | -0.01 (.559) | -0.01 (.604) |
| *f*_o_*_-_SD* | -0.04 (.442) | 0.01 (.875) | 0.01 (.997) | 0.04 (.563) | 1.08 (.591) | 0.99 (.923) | 0.01 (.742) | 0.02 (.401) |
| *D_f_* | 0.06 (.181) | 0.02 (.596) | -0.04 (.550) | -0.06 (.266) | 1.06 (.680) | 1.03 (.844) | 0.01 (.510) | 0.01 (.521) |
| *P_f_* | -0.15 (.005) | -0.08 (.098) | -0.13, (.052) | -0.04 (.488) | 0.92 (.599) | 1.09 (.652) | -0.02 (.246) | -0.02 (.233) |
| Height |  | 0.02 (.702) |  | 0.01 (.960) |  | 1.34 (.080) |  | 0.01 (.885) |
| Weight |  | -0.01 (.832) |  | 0.16 (.040) |  | 0.87 (.412) |  | -0.01 (.765) |
| Age |  | 0.16 (<.001) |  | -0.04 (.460) |  | 0.31 (<.001) |  | -0.02 (.117) |
| Years active |  | 0.42 (<.001) |  | 0.46 (<.001) |  | 2.34 (<.001) |  | 0.10 (<.001) |
| *R^2^* | 0.12 | 0.88 | 0.01 | 0.25 | 0.01 | 0.28 | 0.01 | 0.28 |

**Table S3.** Results of micro-macro multi-level models testing the effect of acoustic measures on measures of fighting success among male MMA fighters.

Note. Results are reported as effect size (p-value). Effect sizes are beta-weights, except for retirment status, which is odds ratio. In all models, variance inflation factors (VIF) were < 1.5, except for those for height and weight (VIFs < 2.5). DV = dependent variable; *f*_o_ = fundamental frequency; *f*_o_-SD *= ­*Variability in fundamental frequency; *D_f_* = formant dispersion; *P_f_* = formant position.


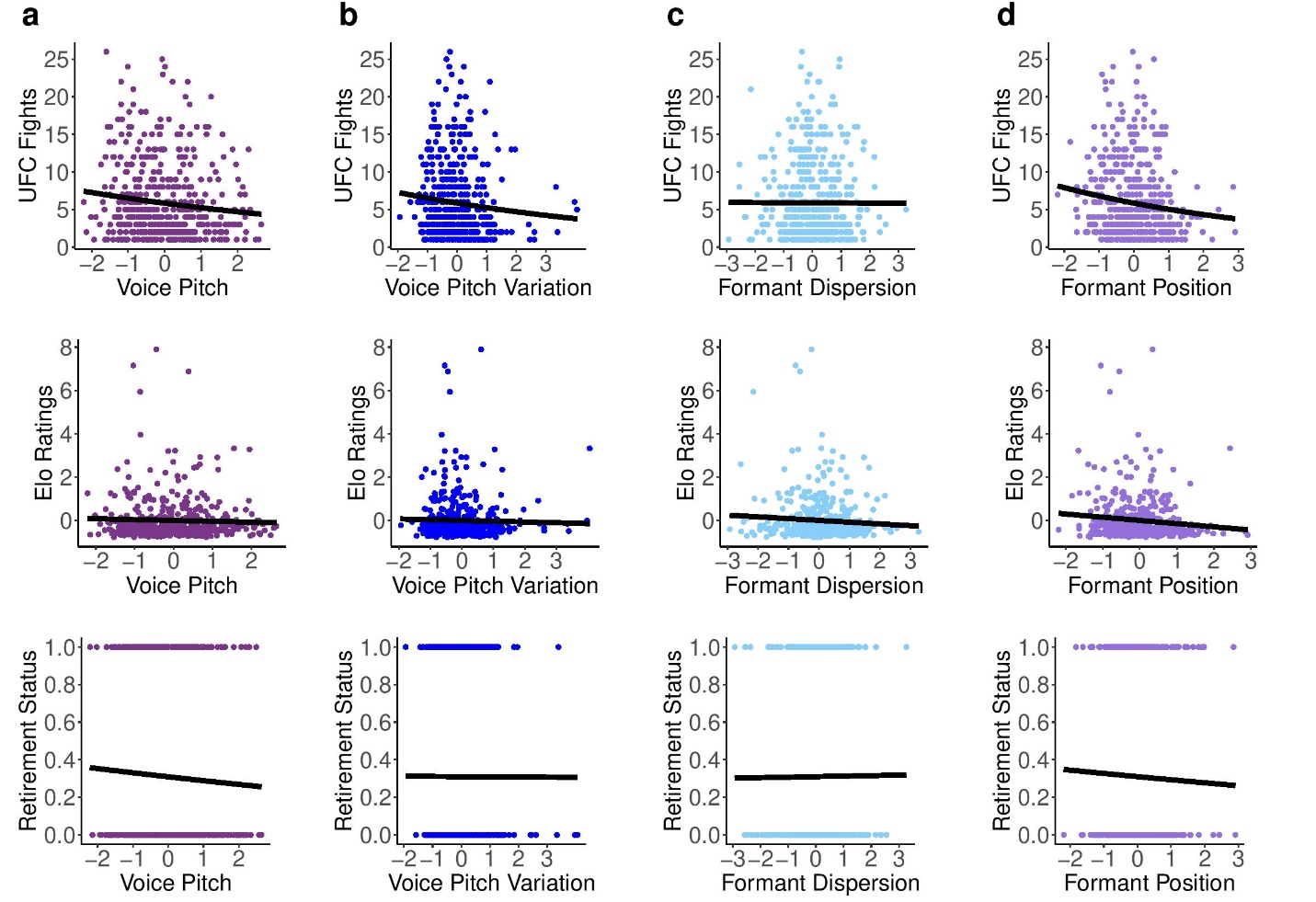


**Figure S1.** Relationship between individual male voice pitch (column a)*_,_* voice pitch variation (column b), formant dispersion (column c), and formant position (column d) and total number of fights (top row), Elo ratings (middle row), and retirement-status (bottom row) among MMA fighters. Regression lines represent best-fit lines for total number of fights in poisson models, Elo ratings in linear models, and retirement-status in binomial models.


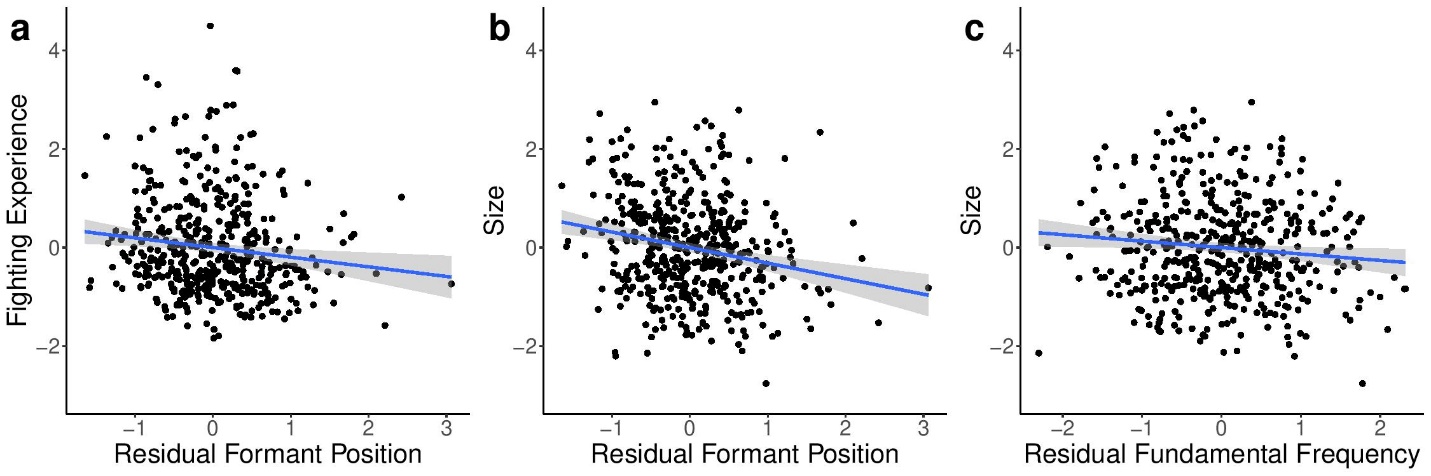


**Figure S2**. Relationship between acoustic measures and components of fighting ability. Formant position predicts Fighting Experience (a) and Fighting Success (b), and fundamental frequency predicts Fighting Success (c) among male MMA fighters. Note. *_­­_*_­_Residual formant position and fundamental frequency are residuals after these acoustic parameters were regressed against other acoustic measures.

**Supplemental Procedures**

Most common multilevel models model macro-micro conditions where higher-level (level 2) explanatory variables are used to predict a lower-level (level 1) outcome variable^69^. Our acoustic measures were collected multiple times (level 1) for each fighter, but our dependent variables: total number of fights, retirement status, and Elo ratings were collected at the fighter level (level 2). In our pre-registration, we planned to predict dependent variables (level 2) using acoustic measures (level 1) via “lme4” and “lmerTest” packages. Nevertheless, we later discovered that this approach violates the assumption of macro-micro conditions in multilevel models, and multi-level models which predict level 2 variables from level 1 variables produce statistically biased results^70^. The “MicroMacroMultilevel” package^71^ circumvented these statistical biases by producing the best linear unbiased predictors (BLUPs) for all group aggregates of variables measured at the lowest level. Hence, we still conducted the pre-registered analyses, but used “MicroMacroMultilevel” package instead of lme4 and lmerTest packages.

Data collection was completed before we finalized our pre-registration document. Before performing any analyses, we inspected our acoustic data to remove any outliers or other data points that were likely to reflect measurement error. Acoustic parameters such as *f*_o_ are highly sensitive to emotional activation as well as physical exhaustion, and our objective was to measure acoustic parameters across all fighters during relaxed, “habitual” speech. To avoid acoustic data captured during physical exhaustion or emotional activation, we identified the recording with the lowest mean *f*_o_ for each fighter. We then eliminated data from recordings in which the mean *f*_o_ was < 20Hz above that value. In addition, we eliminated recordings with mean *f*_o_ < 166 Hz, which previous research^10^ indicates is approximately 4 SD above the adult male mean *f*_o_. We also removed one fighter from our sample whose *P_f_* value was 7.5 SD above the mean.

Among subsets of our recordings where we coded pre- (*n* = 191) vs. post-fight recordings (*n* = 82), we conducted *t*-tests to examine whether acoustic measures differ between the two conditions. No significant differences in *f*_o_ (*t* = 1.70, *p* = .09), *f*_o_*-SD* (*t* = 0.18, *p* = .86)*, D_f_* (*t* = -0.78, *p* = .44)*_,_* or *P_f_* (*t* = -1.62, *p* = .11) were observed between pre- and post-fight recordings. In addition, the use of unbiased linear estimates across recordings for each fighter in all our analyses would further eliminate any potential confounds due to differences in recording conditions.

The raw data and scripts for all our models are also made available online at https://osf.io/md6wj/?view_only=81cf6446a90448a594e1e1ec6b25ce59
